# Prevalence of urinary incontinence in postpartum women and physiotherapy interventions applied: An integrative review

**DOI:** 10.1002/ijgo.15950

**Published:** 2024-10-21

**Authors:** Gifty Koomson, Siyabulela Eric Mgolozeli, Nombeko Mshunqane

**Affiliations:** ^1^ Department of Physiotherapy, School of Healthcare Sciences, Faculty of Health Sciences University of Pretoria Pretoria South Africa; ^2^ Department of Health Studies, School of Social Sciences, College of Human Sciences University of South Africa Pretoria South Africa

**Keywords:** experiences, pelvic floor muscle training, physiotherapy, postpartum women, pregnancy, prevalence, quality of life, urinary incontinence

## Abstract

**Objective:**

This integrative review identified studies that reported the prevalence of physiotherapeutic interventions for urinary incontinence among postpartum women.

**Methods:**

This is an integrative literature review study. We used the integrative literature review framework proposed by Whittemore and Knafl to search for relevant literature.

**Search Strategy:**

The search strategy for electronic databases was developed from the research question and definitions of key concepts, assisted by the librarian. Databases that were searched include Google Scholar, Medline (PubMed), CINAHL, and the Joanna Briggs Institute databases. Both qualitative and quantitative studies that met the inclusion criteria were included. We used the CASP tool to assess the quality of selected papers.

**Data Collection and Analysis:**

The included articles were thematically analyzed. Thirty‐six papers met the inclusion criteria for the review. Six themes emerged from the analysis: prevalence of postpartum UI; risk factors for postpartum UI; antenatal pelvic floor muscle training; conservative treatment and quality of life; experiences of postpartum women with UI; and possible coping strategies adopted by women. Most of the articles were quantitative studies (80.5%); 16.6% were qualitative and 2.7% adopted mixed methods.

**Conclusions:**

Urinary incontinence is common in postpartum women. Antenatal pelvic floor muscle training is protective against postpartum UI and should be the first‐line treatment option.

## Synopsis

Urinary incontinence (UI) is prevalent among women who have given birth, and antenatal pelvic floor muscle training is a first‐line treatment for the prevention of postpartum UI.

## 1 INTRODUCTION

### 1.1 Background

Postpartum urinary incontinence (UI) is the involuntary partial or full release of a woman's bladder after pregnancy and childbirth.^1^ Urinary incontinence markedly affects women's physical, psychological, and social well‐being, and reduces their overall quality of life (QoL).^2^ In the general population, between 26% and 45% of women experience UI^3^, ^4^, ^5^ while about 21%–45% of pregnant women experience UI during their pregnancy.^6^ Stress UI (SUI) and urge UI (UUI) are particularly common during pregnancy, with SUI affecting about 10%–39% of women.^7^, ^8^

Urinary incontinence has been linked to certain risk factors, including age, obesity, and parity, mode of delivery, smoking, drug use, and sedentary lifestyle.^9^, ^10^, ^11^ Handa et al.^12^ posited that SUI and UUI are more common after vaginal birth than after cesarean section (CS), while women who have had a CS may be more likely to experience postpartum UI than nulliparous women.^13^, ^14^ In a multi‐ethnic Norwegian study, it was reported that 41.7% of women experienced UI, with women of African descent having a significantly lower prevalence of UI than women from the Middle East, Eastern and Southeast Asia, and Europe or North America.^15^ In Zaria, north‐western Nigeria, 21% of women attending antenatal clinics experienced UI.^16^ In Ghana, functional incontinence (48.2%) and SUI (43.4%) were the most common types of UI, with about 70% of the 200 women having previously delivered a baby.^17^ A more recent study from Ghana reported a lower prevalence of UI (12%), associated with gravidity, parity, and vaginal deliveries.^18^

To cope with UI, most women go to the toilet more frequently and use pads rather than trying to resolve the underlying cause of their UI symptoms.^19^ Women from India reportedly coped with their UI by restricting their fluid intake (43%), urinating more frequently (45%), and using pads (10%).^20^ Most Indian women restricted their participation in outdoor events (66%) and 5% eventually resorted to self‐medication.^20^ Physiotherapist‐led pelvic floor muscle training (PFMT) has been recommended as a cost‐effective and easily available tool to manage UI.^21^, ^22^ Some studies have also reported the use of collagen injections,^23^ whilst pessaries, vaginal cones, urethral plugs, disposable intravaginal devices, electrical stimulation as well as surgery have been suggested in severe cases.^24^, ^25^

Coping with UI is necessary to avoid the negative effects of the condition. In Sweden, UI had a negative impact on the psychological well‐being of women.^26^ Other studies have reported anxiety, depression, deterioration in sexual life and reduced physical activity.^27^ Mothers with newborn babies are also known to struggle with the negative impacts of UI.^28^, ^29^ According to the International Continent Society, the personal and social problems caused by UI can be understood by examining both subjective experiences and objective data.^30^ In African countries, strong sociocultural beliefs encourage vaginal delivery, which may predispose women to UI. This literature review examines and synthesizes the existing literature on the prevalence, management, and strategies used by postpartum women to cope with UI.

Many efforts have been made to improve the QoL of life of women with UI; however, some people believe that these efforts should include a better understanding of how women are affected by UI.^2^, ^31^ In Turkey, women with UI during the postpartum period experienced problems in their daily lives coupled with emotional and baby‐related effects.^32^ Women who are affected by UI also have few opportunities to share their problems and often feel stigmatized, which prevents them from seeking treatment.^2^ Also, there is a paucity of literature focusing on physiotherapy management strategies for urinary incontinence in postpartum women. This suggests a growing need to identify and summarize information regarding UI among postpartum women.

### 1.2 Objectives

The study sought to examine and synthesize the existing literature on the prevalence, management, and strategies used to cope with UI by postpartum women.

## 2 METHODS

This is an integrative review of the literature.

### 2.1 Problem identification

Many efforts have been made to improve the QoL of life of women with UI; however, some people believe that these efforts should include a better understanding of how women are affected by UI.^2^, ^31^ Women who are affected by UI also have few opportunities to share their problems and often feel stigmatized, which prevents them from seeking treatment.^2^ This suggests a growing need to identify and summarize the literature regarding UI among postpartum women, through the following research questions:

1. What is the prevalence of UI in postpartum women?
2. Which strategies do postpartum women adopt to cope with UI?
3. What are the physiotherapy interventions available for UI in postpartum women?

### 2.2 Search strategy

We used the integrative review framework proposed by Whittemore and Knafl.^33^ This model comprises five stages, which include problem identification, literature search, data evaluation, data analysis, and presentation.^33^ We searched online databases, including Google Scholar, PubMed, Medline, CINAHL, and the Joanna Briggs Institute. Searches were conducted using the keywords “urinary incontinence”, “postpartum women”, “qualitative” and “quantitative research”, “and physiotherapy.” Initially, we searched the literature using individual keywords and later combined the keywords using the Boolean operators “AND” and “OR” to refine the search. The following MESH terms were also considered: gestation, pregnancy, postpartum, antenatal, urine leakage, expectant mothers, postnatal, and puerperium.

The researcher screened the abstracts of the identified articles to select cohort, experimental, and observational studies that fit the inclusion criteria as follows: (1) published between 2001 and 2021; (2) articles published in peer‐reviewed healthcare and public health journals; (3) available full‐text articles detailing the prevalence and management strategies for postpartum UI, both qualitative and quantitative studies; (4) articles published in English; and (5) articles reporting research conducted on adults between 18 and 40 years old. The search included articles globally and narrowed to studies conducted in the African context.

We excluded articles that focused on UI among men or UI which was not related to postpartum women.

Our initial search identified a recently published systematic review^34^ which synthesized evidence on possible associations between parity, UI during pregnancy, and during the first year postpartum. In this study, increasing parity was associated with UI in the first year postpartum,^34^ but there was no conclusive evidence on the risk factors associated with UI. We conducted a second search using all identified keywords and index terms across all included databases (Medline and CINAHL). This search was conducted between November 22 and December 1, 2021. As suggested by Cooke et al. (2012),^35^ only keywords that related to the “phenomenon of interest” were applied to ensure all relevant studies were identified.

### 2.3 Selection criteria

**FIGURE 1:**
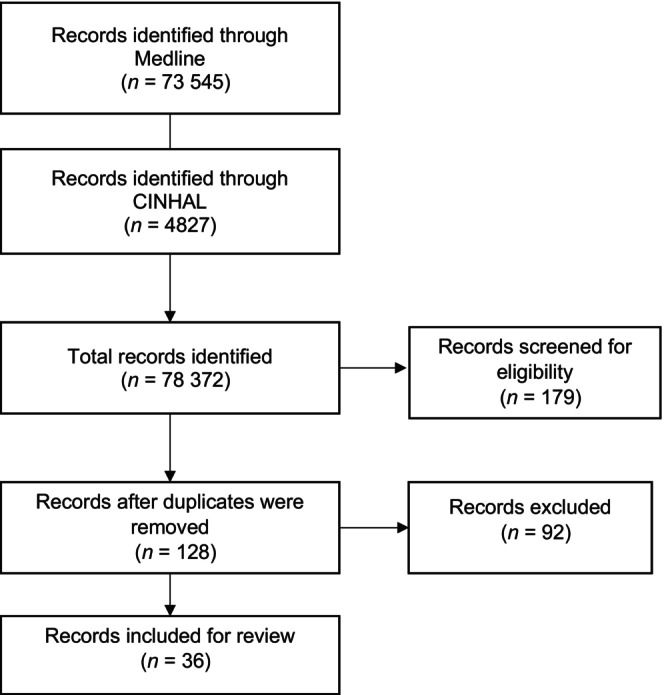
Search strategies used to retrieve articles on urinary incontinence in postpartum women.

Initially, we retrieved 73, 545 articles from Medline and 4827 articles from CINHAL. From these, we extracted 179 articles that met our criteria for date of publication, language, and full text availability. We screened the titles and abstracts of these 179 articles for eligibility and were left with 128 articles, which we assessed for methodological validity using the CASP tool. Ninety‐two articles were excluded based on methodological quality appraisal items. Only 36 articles met our criteria for methodological quality (Figure 1). We assessed the methodological validity of any systematic review papers using the JBI Meta‐Analysis of Statistics Assessment and Review Instrument.^36^ Endnote was used for data management. From the selected articles, we extracted data including specific details about postpartum UI based on epidemiology, coping strategies, management, and experiences of women with postpartum UI. The characteristics of the retrieved articles and their outcomes are presented in Tables 1 and 2, respectively.

**TABLE 1:** Characteristics of the selected studies on urinary incontinence (UI) in postpartum women.

Authors	Title	Study design	Sample	Main findings	Context	Study type
Johannessen et al.^37^	Regular antenatal exercise including PFMT reduces UI 3 months postpartum‐ follow‐up of a randomized controlled trial	Randomized controlled trial	722	A moderate‐intensity exercise program including PFMT reduced prevalence of UI at 3 months postpartum in women who were incontinent at baseline Among women who were incontinent at baseline, 44% and 59% were found to be incontinent at 3 months postpartum in the exercise and control groups, respectively	Norway	Quantitative
Patel et al.^38^	Natural history of UI from first childbirth to 30‐months postpartum	Not clear	3001	Primiparous women who report UI before and during pregnancy should be monitored for the continuation or worsening of UI in the first 2–3 years after birth and treatment options discussed	Not clear	Qualitative
Åström et al.^39^	Quality of life in women with UI seeking care using e‐health	Cross‐sectional survey	373	Women who turned to e‐health had a reduced QoL. The severity of leakage had a greater impact on QoL than the type of UI	Sweden	Quantitative
Moossdorff‐Steinhauser et al.^40^	UI 6 weeks to 1 year postpartum: prevalence, experience of bother, beliefs, and help‐seeking behavior	Digital survey	415	57.1% of women in the Netherlands experience UI from 6 weeks to 1 year after childbirth—38% classified their UI as bothersome; 25% sought help, mostly with specialized (pelvic) physical therapists	Netherlands	Quantitative
Sigurdardottir et al.^41^	Can postpartum PFMT reduce urinary and anal incontinence?: An assessor‐blinded randomized controlled trial	Randomized controlled trial	84	Postpartum PFMT decreased the rate of UI at 6 months postpartum and increased muscle strength and endurance	Iceland	Quantitative
Novo et al.^42^	Prevalence and associated risk factors of UI and dyspareunia during pregnancy and after delivery	Cross‐sectional study	6436	The presence of dyspareunia during pregnancy and having a history of episiotomy were associated with the presence of dyspareunia after delivery. Postpartum SUI was associated with mother's history of SUI before pregnancy and during pregnancy as well as having undergone vaginal delivery. The prevalence rates of SUI and dyspareunia postpartum were 20.4% and 23.4%, respectively	Spain	Quantitative
Jose‐Vaz et al.^43^	Can abdominal hypopressive technique (AHT) improve stress urinary incontinence? An assessor‐blinded randomized controlled trial	Randomized controlled trial	90	PFMT was superior to AHT in improving SUI symptoms, QoL and PFM function	Brazil	Quantitative
Marques et al.^44^	Effect of pelvic floor and hip muscle strengthening in the treatment of stress UI: A randomized clinical trial	Randomized clinical trial	47	Compared to PFMT alone, PFM and hip synergic muscle strengthening reduced the frequency of daily urine loss. No difference between PFMT and PFMT combined with hip synergic muscle strengthening in improving strength, perineometry, or QoL	Brazil	Quantitative
Wagg et al.^45^	Women's experiences, beliefs and knowledge of urinary symptoms in the postpartum period and the perceptions of health professionals: a grounded theory study	Grounded theory	15	Women were more affected by how friends and family would react to UI. Accepting symptoms as normal is complex. The belief that urinary symptoms were “normal” after pregnancy was firmly embedded in the cultural experience of childbirth	Hertfordshire (UK)	Qualitative
Wesnes et al.^46^	Delivery parameters, neonatal parameters and incidence of UI 6 months postpartum: a cohort study	Retrospective cohort study	7561	Certain combinations of delivery parameters and neonatal parameters seem to act together and may increase the risk of UI at 6 months postpartum in a synergetic way	Norway	Quantitative
MacArthur et al.^47^	UI persisting after childbirth: extent, delivery history, and effects in a 12‐year longitudinal cohort study	Twelve‐years longitudinal cohort study	7879	UI may persist for up to 12 years in 75% of women. The risk of UI was only reduced with CS if women had no other delivery mode	United Kingdom	Quantitative
Pizzoferrato et al.^48^	Is prenatal urethral descent a risk factor for UI during pregnancy and the postpartum period?	Randomized controlled trial	186	Prenatal urethral descent and UI during pregnancy are risk factors for UI at 1 year postpartum	France	Quantitative
Fritel et al.^49^	Preventing UI with supervised prenatal pelvic floor exercises: A randomized controlled trial	Randomized controlled trial	282	Supervised PFMT was not superior to written instructions in reducing postnatal UI	France	Quantitative
Obioha et al.^50^	Prevalence and predictors of urinary/anal incontinence after vaginal delivery: prospective study of Nigerian women	Longitudinal prospective study	230	Urinary and anal incontinence were common after vaginal delivery at 12.2% and 13.5%, respectively. Predisposing factors for incontinence should be discouraged	Nigeria	Quantitative
Chang et al.^51^	Association of Mode of Delivery With UI and Changes in UI Over the First Year Postpartum	Prospective longitudinal study	330	Vaginal delivery was associated with higher UI prevalence that persisted for 12 months after birth, but there was no association with interference in daily life after 6 weeks postpartum	Taiwan	Quantitative
Glazener et al.^52^	Twelve‐year follow‐up of conservative management of postnatal urinary and fecal incontinence and prolapse outcomes: randomized controlled trial	Randomized controlled trial	747	It was shown in this study the that initial benefits of a brief nurse‐led conservative treatment for postnatal UI did not persist, about four‐fifths of women with UI 3 months after childbirth still presented with this problem 12 years later	United Kingdom New Zealand	Quantitative
Ahlund et al.^53^	Is home‐based PFMT effective in treatment of urinary incontinence after birth in primiparous women? A randomized controlled trial	Randomized controlled trial	82	The results of this study showed that “home‐based PFMT is effective. However, written training instructions were as efficient as home‐based training with follow‐up visits every 6 weeks”	Sweden	Quantitative
Delarmelindo et al.^54^	Between suffering and hope: rehabilitation from UI as an intervening component	Grounded theory	18	Brazilian women experience UI similar to women in other countries. Women continue to suffer with UI, despite technical and scientific advances, due to limited access to prevention and rehabilitation services	Brazil	Qualitative
Delarmelindo et al.^55^	Women's strategies for coping with UI	Grounded theory	18	Women were found to adopt palliative strategies to avoid being wet. These strategies include: quitting social, leisure and spiritual activities for a long time, attending places with toilets available, using urine collectors, stopping antihypertensives when leaving home, reducing fluid intake, taking sedatives in order to sleep, being careful with the choice of clothes and frequency of exchanges, avoiding coughing, sneezing and laughing and abstaining from sexual activity. These strategies affect human needs and therefore risks to QoL	Brazil	Qualitative
Gyhagen et al.^56^	The prevalence of UI 20 years after childbirth: a national cohort study in singleton primiparae after vaginal or cesarean delivery	A national cohort study	5236	20 years after one birth, vaginal delivery was associated with a 67% increased risk of UI, and UI beyond 10 years increased by 275% compared to CS	Sweden	Quantitative
Hilde et al.^57^	Postpartum PFMT and UI: a randomized controlled trial	Randomized controlled trial	175	Postpartum PFMT did not decrease UI prevalence at 6 months after delivery in primiparous women. Major levator ani muscle defects had no influence	Norway	Quantitative
Macleod et al.^58^	Morbidity experienced by women before and after operative vaginal delivery: prospective cohort study nested within a two‐centre randomized controlled trial of restrictive versus routine use of episiotomy	Longitudinal prospective cohort study	Not clear	Morbidities usually associated with operative vaginal delivery (OVD) may be present after birth, irrespective of operative procedure. Restricted use of episiotomy during OVD may increase rates of urinary morbidity, especially SUI and perineal pain immediately after birth	United Kingdom	Quantitative
Hermansen et al.^19^	Women's explanations for UI, their management strategies, and their quality of life during the postpartum period	Cross‐sectional survey	75	Women are profoundly impacted by UI. Postpartum women's use of ineffective strategies to manage UI. Healthcare clinicians need to actively screen for and treat this condition	Denmark	Quantitative
Boyles et al.^59^	Effect of mode of delivery on the incidence of UI in primiparous women	Population‐based survey	15 787	UI is common immediately postpartum after a first pregnancy. Vaginal delivery increases the risk of UI. Labor and pushing alone, ending in CS does not appear to increase the UI significantly	USA (Oregon)	Quantitative
Griffiths et al.^60^	Group versus individual sessions delivered by a physiotherapist for female UI: an interview study with women attending group sessions nested within a randomized controlled trial	Randomized controlled trial	22	Women are embarrassed by UI and hesitate to attend group therapy. Understanding the content of group sessions will enable women to benefit from group physiotherapy	United Kingdom	Qualitative
Hemachandra et al.^61^	A “usual occurrence”: Stress incontinence among reproductive aged women in Sri Lanka	Descriptive cross‐sectional study	1718	Women are reluctant to seek advice on managing UI. Health providers should pay greater attention to the condition and introduce appropriate preventive measures for women	Sri Lanka	Mixed methods
Wesnes et al.^62^	The effect of UI status during pregnancy and delivery mode on incontinence postpartum. A cohort study	Cohort study	12 679	UI status during pregnancy was not associated with UI postpartum and mode of delivery	Norway	Quantitative
Lee et al.^63^	Prevalence of UI in Korean women: results of a National Health Interview Survey	Explorative, descriptive using interviews	13 484	UI prevalence in women >19 years = 24.4%.SUI most prevalent. Women with UI hesitant to seek medical treatment	Korea	Qualitative
Glazener et al.^64^	New postnatal UI: obstetric and other risk factors in primiparae	Questionnaire survey	3405	Women have less UI after a first delivery by CS. Risk factors for UI: older maternal age, high maternal BMI, heavier babies	United Kingdom New Zealand	Quantitative
Viktrup et al.^65^	Risk of SUI 12 years after the first pregnancy and delivery	Longitudinal, cohort study	241	Onset of SUI during first pregnancy or puerperal period carries an increased risk of long‐lasting symptoms	Denmark	Quantitative
Dumoulin et al.^66^	Physiotherapy for persistent postnatal SUI: a randomized controlled trial	Randomized controlled trial	62	Multimodal supervised pelvic floor physiotherapy is an effective treatment for persistent postnatal SUI	Canada	Quantitative
Mørkved et al.^67^	PFMT during pregnancy to prevent UI: a single‐blind randomized controlled trial	Single‐blind randomized controlled trial	301	Intensive PFMT during pregnancy prevents UI during pregnancy and after delivery, as well as improved strength of the pelvic floor muscle	Trondheim (Norway)	Quantitative
Chiarelli and Jill^68^	Promoting urinary continence in women after delivery: randomized controlled trial	Prospective randomized controlled trial	676	The intervention promotes reduced the prevalence of UI after childbirth. UI became less severe	Australia	Quantitative
Reilly et al.^69^	Prevention of postpartum SUI in primigravidae with increased bladder neck mobility: a randomized controlled trial of antenatal pelvic floor exercises	Single‐blind randomized controlled trials	260	Antenatal supervised pelvic floor exercises reduce the risk of postpartum SUI in primigravidae with bladder neck mobility	United Kingdom	Quantitative
Glazener et al.^70^	Conservative management of persistent postnatal urinary and fecal incontinence: randomized controlled trial	Randomized controlled trial	747	33% of women may have some UI at 3 months after childbirth. Conservative management may reduce the likelihood of urinary and coexisting fecal incontinence persisting 12 months postpartum	New Zealand/ United Kingdom	Quantitative
Meyer et al.^71^	Pelvic floor education after vaginal delivery	Not clear	107	Pelvic floor education, begun 2 months postpartum, significantly reduced the incidence of SUI, but not fecal incontinence or weak pelvic floor	Switzerland	Quantitative

Abbreviations: AHT, abdominal hypopressive technique; CS, cesarean section; PFMT, pelvic floor muscle training; QoL, quality of life; SUI, stress urinary incontinence.

**TABLE 2:** Themes identified from the retrieved articles on urinary incontinence (UI) in postpartum women.

Authors	Prevalence	Risk factors	Antenatal PFMT	Conservative treatment and QoL	Experiences	Coping strategies
Ahlund et al.^53^			✓			
Åström et al.^39^				✓		
Boyles et al.^59^		✓				
Chang et al.^51^	✓	✓				
Chiarelli et al.^68^			✓			
Delarmelindo et al.^54^					✓	
Delarmelindo et al.^55^						✓
Dumoulin et al.^66^			✓			
Fritel et al.^49^			✓			
Glazener et al.^52^						
Glazener et al.^64^		✓				
Glazener et al.^70^		✓				
Griffiths et al.^60^					✓	
Gyhagen et al.^56^	✓	✓				
Hemachandra et al.^61^					✓	
Hermansen et al.^19^						✓
Hilde et al.^57^			✓			
Johannessen et al.^37^			✓			
Jose‐Vaz et al.^43^				✓		
Lee et al.^63^	✓					
MacArthur et al.^47^	✓	✓				
Macleod et al.^58^		✓				
Marques et al.^44^						
Meyer et al.^71^						
Moossdorff‐Steinhauser et al.^40^	✓				✓	
Mørkved et al.^67^			✓			
Novo et al.^42^		✓				
Obioha et al.^50^	✓	✓				
Patel et al.^38^		✓				
Pizzoferrato et al.^48^		✓				
Reilly et al.^69^			✓			
Sigurdardottir et al.^41^			✓			
Viktrup et al.^65^	✓	✓				
Wagg, Kendall^45^					✓	
Wesnes et al.^46^		✓				
Wesnes et al.^62^	✓	✓				
Overall	8/36	14/36	9/36	2/36	5/36	2/36

Abbreviation: PFMT, pelvic floor muscle training.

### 2.4 Data collection and analysis

During the data collection, ethical considerations were followed. In effect, we adhered to all ethical standards set out for studies without direct contact with human or animal subjects. The study was granted ethical clearance by the University of Pretoria, Faculty of Health Sciences Research Ethics Committee (Ref: 491/2021).

The characteristics of the included studies, including authors, title, study design, context, and study type, are summarized in Table 1. Using the CASP tool, we observed that close to 50% of the retrieved articles followed rigorous methodological principles. Most of the articles were quantitative (80.5%, *n* = 29), while 6 (16.6%) were qualitative and 1 (2.7%) was a mixed‐methods study. Twelve papers were randomized controlled trials.

With regards to context, 24 articles were conducted in European countries. Three were conducted in Asian countries. One article reported on research conducted in Nigeria (Africa) and another one on research conducted in Australia. One article was a qualitative study without a clearly described study design or setting.^38^

## 3 RESULTS

All selected records were coded and thematically analyzed.^72^ Six themes emerged from the retrieved articles, namely: (1) prevalence of UI in postpartum women; (2) risk factors for postpartum UI; (3) antenatal pelvic floor muscle training; (4) conservative treatment and QoL; (5) experiences of postpartum women with UI; and (6) coping strategies.

The first theme entails the prevalence of postpartum UI and, from this theme, it was found that eight articles reported that the prevalence of UI was high among postpartum women.^40^, ^47^, ^50^, ^51^, ^56^, ^62^, ^63^, ^65^ The incidence of UI increases after childbirth. More than 50% of all postpartum women in the Netherlands experience UI from 6 weeks to 1 year after giving birth.^40^

In the second theme, it was revealed that 14 articles described possible risk factors for postpartum UI.^38^, ^42^, ^46^, ^47^, ^48^, ^50^, ^51^, ^56^, ^58^, ^59^, ^62^, ^64^, ^65^, ^70^ Risk factors include older maternal age, higher body mass index (BMI) of mothers, heavier babies, and urinary incontinence starting during pregnancy.^64^

Theme 3 deals with antenatal pelvic floor muscle training and from this theme, nine articles reported on the effect of PFMT on the prevalence of UI.^37^, ^41^, ^49^, ^53^, ^57^, ^66^, ^67^, ^68^, ^69^ In support of PFMT, Johannessen et al.^37^ reported that moderate‐intensity antenatal exercise programs that include PFMT protected against UI in the early postpartum period, especially in women with existing UI.^37^ Seven articles advocated for PFMT in the treatment of UI^37^, ^41^, ^53^, ^66^, ^67^, ^68^, ^69^ and two articles suggested that PFMT did not significantly reduce the symptoms of UI.^49^, ^57^

For theme 4, two quantitative studies, one from Brazil and the other from Sweden, reported that conservative management of UI improves the QoL of women with UI.^39^, ^43^ When treated, the symptoms of UI become less severe, which improves the QoL.^39^ Ten articles explored various conservative treatment methods for UI. A randomized controlled trial reported that abdominal hypopressive techniques (AHTs) combined with PFMT improved urinary symptoms, but that PFMT was superior to AHT.^43^ Another quantitative study reported that pelvic floor education beginning 2 months postpartum significantly reduced the incidence of SUI.^71^ According to Dumoulin et al.,^66^ multimodal supervised pelvic floor physiotherapy can effectively treat persistent postnatal SUI. Four studies suggested a need for proper avenues to address UI issues among postpartum women.^19^, ^39^, ^45^, ^61^

A Danish study recommended that healthcare clinicians should actively screen and treat UI.^19^ Similarly, Wagg et al.^45^ recommended that referral must be initiated in general practice and that all healthcare practitioners should be aware of the guidelines, available services, and treatment for UI.^45^ Åström et al.^39^ further suggested that women should be individually assessed to establish the severity of symptoms and subsequent intervention.^39^

In theme 5, which deals with the experiences of postpartum women with UI, it was revealed that five articles described the experiences of women with UI^40^, ^45^, ^54^, ^60^, ^61^; of these five studies, three were conducted in Europe, and two were from Asia and South America. Three articles reported on the experiences of postpartum women with UI,^40^, ^45^, ^61^ while the other two reported on the experiences of women with UI in general.^54^, ^60^ One study reported that women were prepared to lie about their UI symptoms to avoid examination.^45^ In the Netherlands, more than half of all postpartum women from 6 weeks to 1 year experience UI (57.1%), with many of these women (38%) classifying their UI symptoms as bothersome.^40^

Theme 6 has to do with possible coping strategies adopted by women. Two articles, one from Europe and the other from South America, reported on coping strategies adopted by women.^19^, ^55^ In Brazil, a qualitative study reported that women largely adopted palliative strategies to avoid being wet.^55^ These palliative strategies included quitting social, leisure, and spiritual activities, only going to venues that had toilets, using urine collectors, stopping antihypertensives when leaving home, avoiding coughing, sneezing, and laughing, and abstaining from sexual activity.^55^ Similarly in Sweden, women resorted to going to the toilet more frequently (64%) and using pads (56%).^19^

In this review, we identified and synthesized literature on the epidemiology, coping strategies, management, and experiences of postpartum women living with UI. We retrieved 36 papers, which dealt with six themes. We weighted the importance of themes based on the number of papers that dealt with each theme. Most of the literature on UI in postpartum women dealt with identifying risk factors for UI, as well as identifying strategies for managing UI. Most of the studies that reported the prevalence of UI reported a high prevalence of UI among women who gave vaginal birth. A population study in Sweden reported that UI and overactive bladder are highly prevalent conditions in women.^73^ Recent studies show that in Turkey, about 87.2% of women with UI have mentioned that it affects their quality of life. However, less than 15% of these women managed to seek medical help.^74^ Other studies show that most women do not seek medical help because of embarrassment or stigmas associated with this disease, mostly influenced by culture.^74^

Urinary incontinence is strongly associated with giving vaginal birth. In our review, 38% of articles investigated the risk factors of postpartum UI. In some countries, trying to avoid UI by choosing CS may be quite a controversial decision because vaginal births have so many other benefits. Radiological studies and epidemiological literature confirm that pelvic floor injury following vaginal birth as well as pelvic organ prolapse contribute to UI.^75^ The literature mentions several factors that contribute to the development of UI among postpartum women, including episiotomy, increasing maternal age, BMI, pregnancy‐related hormonal changes, the pressure of the growing uterus, neonatal birth weight and severe vaginal tears, instrumental delivery, epidural or spinal anesthesia, and pre‐pregnancy BMI.^58^, ^64^, ^76^, ^77^ These factors could be used to develop a checklist for identifying at‐risk mothers.

The second most important theme involved managing postpartum UI. Paramount among the interventions available for the management of UI is the performance of PMFT; the main aim of PFMT is to improve pelvic floor muscle function. Usually, PFMT is performed to increase strength, endurance, and muscle coordination.^22^

A systematic review conducted in Brazil among adult women concluded that the management of UI should be personalized and customized, to meet the preferences and expectations of people affected by UI.^78^ Understanding how women experience postpartum UI may help to develop appropriate preventative measures. A qualitative study reported that women were reluctant to seek advice,^61^ as they are influenced by their cultural and religious backgrounds.^78^ Women may even go so far as lying to their healthcare providers about their UI symptoms just to avoid being examined.^45^ It is imperative that proper avenues to address women's UI issues are created as was suggested by three of the papers reviewed.^19^, ^45^, ^61^

## 4 CONCLUSION

This integrative literature review adds to the available literature on postpartum UI by integrating evidence regarding postpartum women with this condition. Recent literature suggests that UI is prevalent, ranging from 27% to 33% among women who have given birth, especially vaginal birth. Several studies have indicated that antenatal PFMT is protective against postpartum UI and could be regarded as first‐line treatment for UI. Three of the themes identified in this review may require further qualitative exploration namely “experiences of postpartum women with UI,” “conservative treatment is capable of improving QoL,” and “possible coping strategies adopted by women.” More research is required in low‐ and middle‐income countries.

Based on the conclusions drawn, the study provides implications and recommendations. Appropriate channels are needed to address issues concerning women with postpartum UI. Healthcare providers need to be aware of the phenomenon. Qualitative studies may provide more insight into how postpartum women experience UI. From this review, it is clear that more evidence is needed from the sub‐Saharan African region.

## AUTHOR CONTRIBUTIONS

Gifty Koomson was responsible for data collection, analysis, and manuscript writing. Nombeko Mshunqane was responsible for the review of data and the final restructuring of the manuscript. Siyabulela Eric Mgolozeli was responsible for the research design and general review of the data.

## FUNDING INFORMATION

The study received no external funding.

## CONFLICT OF INTEREST STATEMENT

The authors have no conflicts of interest.

## Supporting information


**Data S1:** PRISMA_2020_checklist.

## Data Availability

Data will be available on request from the authors. The data that support the findings of this study are available from the corresponding author upon reasonable request.

## References

[1] Brusie C . Postpartum incontinence causes and treatment. 2019, Accessed May 10, 2021 https://www.verywellfamily.com/postpartum‐incontinence‐4580237

[2] Higa R , de Rivoreˆdo CC L , de Moraes Lopes M , et al. Life experiences of Brazilian women with urinary incontinence. Texto Context Enferm. 2010;19:627‐635.

[3] Demirci N , Aba Y , Süzer F . Urinary incontinence and its effects on life quality for women over 18. Fırat Health Serv J. 2012;7:23‐37.

[4] Zumrutbas AE , Bozkurt AI , Alkis O , Toktas C , Cetinel B , Aybek Z . The prevalence of nocturia and nocturnal polyuria: can new cutoff values be suggested according to age and sex? Int Neurourol J. 2016;20:304‐310. doi:10.5213/inj.1632558.279 28043108 PMC5209574

[5] Baykuş N , Yenal K . Prevalence of urinary incontinence in women aged 18 and over and affecting factors. J Women Aging. 2019;32:578‐590. 31640491 10.1080/08952841.2019.1682923

[6] Haylen BT , de Ridder D , Freeman RM , et al. An international Urogynecological association (IUGA)/international continence society (ICS) joint report on the terminology for female pelvic floor dysfunction. Neurourol Urodyn. 2010;29(4):20. Accessed November 27, 2009. doi:10.1002/nau.20798 19941278

[7] Cerruto MA , D'Elia C , Aloisi A , et al. Prevalence, incidence and obstetric factors' impact on female urinary incontinence in Europe: a systematic review. Urol Int. 2013;90:1‐9. Accesssed August 8, 2012. doi:10.1159/000339929 22868349

[8] Ebbesen MH , Hunskaar S , Rortveit G , et al. Prevalence, incidence and remission of urinary incontinence in women: longitudinal data from the Norwegian HUNT study (EPINCONT). BMC Urol. 2013;13(27). Accessed June 06, 2013. https://bmcurol.biomedcentral.com/articles/10.1186/1471‐2490‐13‐27#citeas 10.1186/1471-2490-13-27 PMC3674916 23721491

[9] Gyhagen M , Åkervall S , Molin M , Milsom I . The effect of childbirth on urinary incontinence: a matched cohort study in women aged 40–64 years. Am J Obstet Gynecol. 2019;221(322):e321‐322. e317. doi:10.1016/j.ajog.2019.05.022 31121136

[10] Jerez‐Roig J , Booth J , Skelton DA , Giné‐Garriga M , Chastin SFM , Hagen S . Is urinary incontinence associated with sedentary behaviour in older women? Analysis of data from the National Health and nutrition examination survey. PLoS One. 2020;15:e0227195. doi:10.1371/journal.pone.0227195 32017767 PMC6999862

[11] Lukacz ES , Santiago‐Lastra Y , Albo ME , Brubaker L . Urinary incontinence in women: a review. JAMA. 2017;318:1592‐1604. doi:10.1001/jama.2017.12137 29067433

[12] Handa VL , Pierce CB , Muñoz A , et al. Longitudinal changes in overactive bladder and stress incontinence among parous women. Neurourol Urodyn. 2015;34:356‐361. Accessed March 19,2014:. doi:10.1002/nau.22583 24633996 PMC4163529

[13] Press J , Klein M , Kaczorowski J , et al. Does cesarean section reduce postpartum urinary incontinence? A Systematic Review. Birth. 2007;34:228‐237. 17718873 10.1111/j.1523-536X.2007.00175.x

[14] Rortveit G , Daltveit AK , Hannestad YS , et al. Urinary incontinence after vaginal delivery or cesarean section. N Engl J Med. 2003;348:900‐907. doi:10.1056/NEJMoa021788 12621134

[15] Bø K , Pauck Øglund G , Sletner L , et al. The prevalence of urinary incontinence in pregnancy among a multi‐ethnic population resident in Norway. BJOG. 2012;119:1354‐1360. doi:10.1111/j.1471-0528.2012.03435.x 22827706

[16] Adaji SE , Shittu OS , Bature SB , Nasir S , Olatunji O . Suffering in silence: pregnant women's experience of urinary incontinence in Zaria, Nigeria. Eur J Obstet Gynecol Reprod Biol. 2010;150:19‐23. doi:10.1016/j.ejogrb.2010.02.008 20189707

[17] Adanu RM , De Lancey JO , Miller JM , et al. The physical finding of stress urinary incontinence among African women in Ghana. Int Urogynecol J Pelvic Floor Dysfunct. 2006;17:581‐585. doi:10.1007/s00192-005-0062-x 16491324

[18] Ofori AA , Osarfo J , Agbeno EK , et al. Prevalence and determinants of non‐fistulous urinary incontinence among Ghanaian women seeking gynaecologic care at a teaching hospital. PLoS One. 2020;15:e0237518. doi:10.1371/journal.pone.0237518 32810136 PMC7433879

[19] Hermansen IL , O'Connell BO , Gaskin CJ . Women's explanations for urinary incontinence, their management strategies, and their quality of life during the postpartum period. J Wound Ostomy Continence Nurs. 2010;37:187‐192. doi:10.1097/WON.0b013e3181cf7946 20228661

[20] Seshan V . Coping strategies and sel measures adopted by the women with urinary incontinence and its effects on QOL. Obstet Gynecol Int J. 2016;5:465. doi:10.15406/ogij.2016.05.00187

[21] Shamliyan TA , Kane RL , Wyman J , et al. Systematic review: randomized, controlled trials of nonsurgical treatments for urinary incontinence in women. Ann Intern Med. 2008;148:459‐473. doi:10.7326/0003-4819-148-6-200803180-00211 18268288

[22] Dumoulin C , Hay‐Smith J , Habée‐Séguin G , et al. Pelvic floor muscle training versus no treatment, or inactive control treatments, for urinary incontinence in women. Neurourol Urodyn. 2015;34:300‐308. doi:10.1002/nau.22700 25408383

[23] Mohr S , Siegenthaler M , Mueller MD , Kuhn A . Bulking agents: an analysis of 500 cases and review of the literature. Int Urogynecol J. 2013;24:241‐247. doi:10.1007/s00192-012-1834-8 22707004

[24] Hendrix SL , Cochrane BB , Nygaard IE , et al. Effects of estrogen with and without progestin on urinary incontinence. JAMA. 2005;293:935‐948. doi:10.1001/jama.293.8.935 15728164

[25] Steinauer JE , Waetjen LE , Vittinghoff E , et al. Postmenopausal hormone therapy: does it cause incontinence? Obstet Gynecol. 2005;106:940‐945. doi:10.1097/01.Aog.0000180394.08406.15 16260510 PMC1557396

[26] Åhlund S , Rothstein E , Rådestad I , et al. Urinary incontinence after uncomplicated spontaneous vaginal birth in primiparous women during the first year after birth. Int Urogynecol J. 2020;31:1409‐1416. doi:10.1007/s00192-019-03975-0 31139858 PMC7306031

[27] Pizzol D , Demurtas J , Celotto S , et al. Urinary incontinence and quality of life: a systematic review and meta‐analysis. Aging Clin Exp Res. 2021;33:25‐35. doi:10.1007/s40520-020-01712-y 32964401 PMC7897623

[28] Holroyd‐Leduc JM , Straus SE . Management of urinary incontinence in women: scientific review. JAMA. 2004;291:986‐995. doi:10.1001/jama.291.8.986 14982915

[29] Sottner O , Zahumensky J , Krcmar M , et al. Urinary incontinence in a group of primiparous women in The Czech Republic. Gynecol Obstet Investig. 2006;62:33‐37. 16514239 10.1159/000091820

[30] Karl M . The definition, prevalence, and risk factors for stress urinary incontinence. Rev Urol. 2004;6:3‐9. PMC1472862 16985863

[31] Volkmer C , Monticelli M , Reibnitz KS , Brüggemann OM , Sperandio FF . Female urinary incontinence: a systematic review of qualitative studies. Ciênc Saúde Colet. 2012;17:2703‐2715. 10.1590/s1413-81232012001000019 23099757

[32] Erkal Aksoy Y , Akin B , Dereli YS . Postpartum urinary incontinence: a qualitative study on sexuality and life experiences of Muslim Turkish women. Female Pelvic Med Reconstr Surg. 2021;27:514‐520. doi:10.1097/spv.0000000000001072 34074935

[33] Whittemore R , Knafl K . The integrative review: updated methodology. J Advert. 2005;52:546‐553. doi:10.1111/j.1365-2648.2005.03621.x 16268861

[34] Wuytack F , Moran P , Daly D , Begley C . Is there an association between parity and urinary incontinence in women during pregnancy and the first year postpartum?: a systematic review and meta‐analysis. Neurourol Urodyn. 2021;41:54‐90. doi:10.1002/nau.24785 34529861

[35] Cooke A , Smith D , Booth A . Beyond PICO: the SPIDER tool for qualitative evidence synthesis. Qual Health Res 2012;22(10):145‐1443. doi:10.1177/1049732312452938 22829486

[36] Peters M , Godfrey C , Khalil H , et al. Guidance for conducting systematic scopingreviews. Int J Evid Based Healthc. 2015;13:141‐146. 26134548 10.1097/XEB.0000000000000050

[37] Johannessen HH , Frøshaug BE , Lysåker PJG , et al. Regular antenatal exercise including pelvic floor muscle training reduces urinary incontinence 3 months postpartum‐follow up of a randomized controlled trial. Acta Obstet Gynecol Scand. 2021;100:294‐301. doi:10.1111/aogs.14010 32996139

[38] Patel K , Long JB , Boyd SS , Kjerulff KH . Natural history of urinary incontinence from first childbirth to 30‐months postpartum. Arch Gynecol Obstet. 2021;304:713‐724. doi:10.1007/s00404-021-06134-3 34175975

[39] Åström Y , Asklund I , Lindam A , Sjöström M . Quality of life in women with urinary incontinence seeking care using e‐health. BMC Womens Health. 2021;21:337. doi:10.1186/s12905-021-01477-0 34544393 PMC8454026

[40] Moossdorff‐Steinhauser H , Berghmans B , Spaanderman M , Bols EMJ . Urinary incontinence 6 weeks to 1 year post‐partum: prevalence, experience of bother, beliefs, and help‐seeking behavior. Int Urogynecol J. 2021;32:1817‐1824. doi:10.1007/s00192-020-04644-3 33484286 PMC8295159

[41] Sigurdardottir T , Steingrimsdottir T , Geirsson RT , Halldorsson TI , Aspelund T , Bø K . Can postpartum pelvic floor muscle training reduce urinary and anal incontinence?: an assessor‐blinded randomized controlled trial. Am J Obstet Gynecol. 2020;222:247. e241‐247.e248. doi:10.1016/j.ajog.2019.09.011 31526791

[42] Novo R , Perez‐Rios M , Santiago‐Pérez MI , Butler H , Malvar A , Hervada X . Prevalence and associated risk factors of urinary incontinence and dyspareunia during pregnancy and after delivery. Eur J Obstet Gynecol Reprod Biol. 2020;245:45‐50. doi:10.1016/j.ejogrb.2019.10.020 31851895

[43] Jose‐Vaz LA , Andrade CL , Cardoso LC , Bernardes BT , Pereira‐Baldon VS , Resende APM . Can abdominal hypropressive technique improve stress urinary incontinence? An assessor‐blinded randomized controlled trial. Neurourol Urodyn. 2020;39:2314‐2321. doi:10.1002/nau.24489 32813928

[44] Marques SAA , Silveira S , Pássaro AC , et al. Effect of pelvic floor and hip muscle strengthening in the treatment of stress urinary incontinence: a randomized clinical trial. J Manip Physiol Ther. 2020;43:247‐256. doi:10.1016/j.jmpt.2019.01.007 32703614

[45] Wagg AR , Kendall S , Bunn F . Women's experiences, beliefs and knowledge of urinary symptoms in the postpartum period and the perceptions of health professionals: a grounded theory study. Prim Health Care Res Dev. 2017;18:448‐462. doi:10.1017/s1463423617000366 28825530

[46] Wesnes SL , Hannestad Y , Rortveit G . Delivery parameters, neonatal parameters and incidence of urinary incontinence six months postpartum: a cohort study. Acta Obstet Gynecol Scand. 2017;96:1214‐1222. doi:10.1111/aogs.13183 28626856

[47] MacArthur C , Wilson D , Herbison P , et al. Urinary incontinence persisting after childbirth: extent, delivery history, and effects in a 12‐year longitudinal cohort study. BJOG. 2016;123:1022‐1029. doi:10.1111/1471-0528.13395 25846816

[48] Pizzoferrato AC , Fauconnier A , Bader G , de Tayrac R , Fort J , Fritel X . Is prenatal urethral descent a risk factor for urinary incontinence during pregnancy and the postpartum period? Int Urogynecol J. 2016;27:1003‐1011. 26797099 10.1007/s00192-015-2918-z

[49] Fritel X , de Tayrac R , Bader G , et al. Preventing urinary incontinence with supervised prenatal pelvic floor exercises: a randomized controlled trial. Obstet Gynecol. 2015;126:370‐377. doi:10.1097/aog.0000000000000972 26241428

[50] Obioha KC , Ugwu EO , Obi SN , et al. Prevalence and predictors of urinary/anal incontinence after vaginal delivery: prospective study of Nigerian women. Int Urogynecol J. 2015;26:1347‐1354. doi:10.1007/s00192-015-2690-0 25894903

[51] Chang S‐R , Chen K‐H , Lin H‐H , Lin MI , Chang TC , Lin WA . Association of mode of delivery with urinary incontinence and changes in urinary incontinence over the first year postpartum. Obstet Gynecol. 2014;123:568‐577. 24499754 10.1097/AOG.0000000000000141

[52] Glazener CM , MacArthur C , Hagen S , et al. Twelve‐year follow‐up of conservative management of postnatal urinary and faecal incontinence and prolapse outcomes: randomised controlled trial. BJOG. 2014;121:112‐120. doi:10.1111/1471-0528.12473 24148807

[53] Ahlund S , Nordgren B , Wilander EL , Wiklund I , Fridén C . Is home‐based pelvic floor muscle training effective in treatment of urinary incontinence after birth in primiparous women? A randomized controlled trial. Acta Obstet Gynecol Scand. 2013;92:909‐915. doi:10.1111/aogs.12173 23672520

[54] Delarmelindo R , Parada CM , Rodrigues RA , et al. Between suffering and hope: rehabilitation from urinary incontinence as an intervening component. Ciênc Saúde Colet. 2013;18:1981‐1991. doi:10.1590/s1413-81232013000700013 23827902

[55] Delarmelindo R , Parada C , Roddrigues R , et al. Women's strategies for coping with urinary incontinence. Rev Esc Enferm. 2013;47:296‐302. 10.1590/s0080-62342013000200004 23743893

[56] Gyhagen M , Bullarbo M , Nielsen TF , et al. The prevalence of urinary incontinence 20 years after childbirth: a national cohort study in singleton primiparae after vaginal or caesarean delivery. BJOG. 2013;120:144‐151. doi:10.1111/j.1471-0528.2012.03301.x 22413831

[57] Hilde G , Stær‐Jensen J , Siafarikas F , Ellström Engh M , Bø K . Postpartum pelvic floor muscle training and urinary incontinence: a randomized controlled trial. Obstet Gynecol. 2013;122:1231‐1238. doi:10.1097/aog.0000000000000012 24201679

[58] Macleod M , Goyder K , Howarth L , et al. Morbidity experienced by women before and after operative vaginal delivery: prospective cohort study nested within a two‐centre randomised controlled trial of restrictive versus routine use of episiotomy. Int J Obsteric Gynaecol. 2013;120:1020‐1027. doi:10.1111/1471-0528.12184 23464382

[59] Boyles SH , Li H , Mori T , Osterweil P , Guise JM . Effect of mode of delivery on the incidence of urinary incontinence in primiparous women. Obstet Gynecol. 2009;113:134‐141. 19104369 10.1097/AOG.0b013e318191bb37

[60] Griffiths F , Pepper J , Jørstad‐Stein EC , Smith JF , Hill L , Lamb SE . Group versus individual sessions delivered by a physiotherapist for female urinary incontinence: an interview study with women attending group sessions nested within a randomised controlled trial. BMC Womens Health. 2009;9:25. doi:10.1186/1472-6874-9-25 19744315 PMC2753338

[61] Hemachandra NN , Rajapaksa LC , Manderson L . A “usual occurrence:” stress incontinence among reproductive aged women in Sri Lanka. Soc Sci Med. 2009;69:1395‐1401. doi:10.1016/j.socscimed.2009.08.019 19758738

[62] Wesnes SL , Hunskaar S , Bo K , Rortveit G . The effect of urinary incontinence status during pregnancy and delivery mode on incontinence postpartum. A cohort study. BJOG. 2009;116:700‐707. doi:10.1111/j.1471-0528.2008.02107.x 19220234 PMC2675011

[63] Lee K‐S , Sung HH , Na S , Choo MS . Prevalence of urinary incontinence in Korean women: results of a National Health Interview Survey. World J Urol. 2008;26:179‐185. doi:10.1007/s00345-008-0239-2 18265989

[64] Glazener CM , Herbison GP , MacArthur C , et al. New postnatal urinary incontinence: obstetric and other risk factors in primiparae. BJOG. 2006;113:208‐217. doi:10.1111/j.1471-0528.2005.00840.x 16412000

[65] Viktrup L , Rortveit G , Lose G . Risk of stress urinary incontinence twelve years after the first pregnancy and delivery. Obstet Gynecol. 2006;108:248‐254. doi:10.1097/01.AOG.0000226860.01127.0e 16880292

[66] Dumoulin C , Lemieux MC , Bourbonnais D , Gravel D , Bravo G , Morin M . Physiotherapy for persistent postnatal stress urinary incontinence: a randomized controlled trial. Obstet Gynecol. 2004;104:504‐510. doi:10.1097/01.Aog.0000135274.92416.62 15339760

[67] Mørkved S , Bø K , Schei B , et al. Pelvic floor muscle training during pregnancy to prevent urinary incontinence: a single‐blind randomized controlled trial. Obstet Gynecol. 2003;101:313‐319. doi:10.1016/s0029-7844(02)02711-4 12576255

[68] Chiarelli P , Jill C . Promoting urinary incontinence in women after delivery: randomised control trial. BJM. 2002;324:324. doi:10.1136/bmj.324.7348.1241 PMC113274 12028976

[69] Reilly ET , Freeman RM , Waterfield MR , et al. Prevention of postpartum stress incontinence in primigravidae with increased bladder neck mobility: a randomised controlled trial of antenatal pelvic floor exercises. BJOG. 2002;109:68‐76. doi:10.1111/j.1471-0528.2002.t01-1-01116.x 11845813

[70] Glazener CM , Herbison GP , Wilson PD , et al. Conservative management of persistent postnatal urinary and faecal incontinence: randomised controlled trial. BMJ. 2001;323:593‐596. doi:10.1136/bmj.323.7313.593 11557703 PMC55571

[71] Meyer S , Hohlfeld P , Achtari C , et al. Pelvic floor education after vaginal delivery. Obstet Gynecol. 2001;97:673‐677. doi:10.1016/S0029-7844(00)01101-7 11339914

[72] Lester JN , Cho Y , Lochmiller CR . Learning to do qualitative data analysis: a starting point. Hum Resour Dev Rev. 2020;19:94‐106. doi:10.1177/1534484320903890

[73] Milsom I . Lower urinary tract symptoms in women. Curr Opin Urol. 2009;19:337‐341. doi:10.1097/MOU.0b013e32832b659d 19444118

[74] Hammad FT . Prevalence, social impact and help‐seeking behaviour among women with urinary incontinence in the Gulf countries: a systematic review. Eur J Obstet Gynecol Reprod Biol. 2021;266:150‐156. 34653920 10.1016/j.ejogrb.2021.10.005

[75] Panayi DC , Khullar V . Urogynaecological problems in pregnancy and postpartum sequelae. Curr Opin Obstet Gynecol. 2009;8:97‐100. doi:10.1097/GCO.0b013e328321e44b 19125008

[76] Sangsawang B , Sangsawang N . Stress urinary incontinence in pregnant women: a review of prevalence, pathophysiology, and treatment. Int Urogynecol J. 2013;24:901‐912. doi:10.1007/s00192-013-2061-7 23436035 PMC3671107

[77] Siahkal SF , Iravani M , Mohaghegh Z , Sharifipour F , Zahedian M . Maternal, obstetrical and neonatal risk factors' impact on female urinary incontinence: a systematic review. Int Urogynecol J. 2020;31:2205‐2224. doi:10.1007/s00192-020-04442-x 32712698

[78] Mendes A , Hoga L , Gonçalves B , et al. Adult women's experiences of urinary incontinence: a systematic review of qualitative evidence. JBI Database System Rev Implement Rep. 2017;15:1350‐1408. doi:10.11124/jbisrir-2017-003389 28498174

